# Epidemiology of Classical Swine Fever in Japan—A Descriptive Analysis of the Outbreaks in 2018–2019

**DOI:** 10.3389/fvets.2020.573480

**Published:** 2020-09-22

**Authors:** Yumiko Shimizu, Yoko Hayama, Yoshinori Murato, Kotaro Sawai, Emi Yamaguchi, Takehisa Yamamoto

**Affiliations:** Viral Disease and Epidemiology Research Division, National Institute of Animal Health, National Agriculture and Food Research Organization, Tsukuba, Japan

**Keywords:** classical swine fever (CSF), domestic pig (Sus scrofa), epidemiology, outbreak investigation, Japan

## Abstract

This study describes the epidemiological characteristics of classical swine fever (CSF) outbreaks in Japan. The first case was confirmed in September 2018, 26 years after the last known case. Outbreaks occurred on 39 farms, 34 commercial farms, and 5 non-commercial farms, between September 2018 and August 2019. In this study, a descriptive analysis was conducted of the epidemiological data on the characteristics of the affected farms, clinical manifestations, intra-farm transmission, association with infected wild boars, and control measures implemented on the farms. Twenty-eight of the 34 affected commercial farms were farrow-to-finish farms. It was assumed that the major risk factors were frequent human-pig interactions and the movement of pigs between farms. Fever and leukopenia were commonly observed in infected pigs. In 12 out of 18 farms where clinical manifestations among fattening pigs was the reason for notification, death was the most frequent clinical manifestation, but the proportion of dead animals did not exceed 0.5% of the total number of animals at most of the affected farms. Therefore, the clinical form of CSF in Japan was considered to be sub-acute. Twenty-three of the 29 farms (79%) with pigs at multiple stages (i.e., piglets, fattening pigs, and sows), had infection across the multiple stages. Many of these farms were within 5 km of the site where the first infected wild boars had been discovered, suggesting that infected wild boars were the source of infection. Infections still occurred at farms that had implemented measures at their farm boundaries to prevent the introduction of the virus into their farms, such as disinfection of vehicles and people, changing boots of the workers, and installation of perimeter fences. It is necessary to continue to strengthen biosecurity measures for farms located in areas with infected wild boars and to continue monitoring the distribution of infected wild boars so that any abnormalities can be reported and inspected at an early stage.

## Introduction

Classical swine fever (CSF) is among the most devastating contagious diseases in pigs. Due to its impact on pig production, the prevention and control of the disease has been a major priority in pig producing countries. In Japan, 9.2 million heads of pigs are reared at about 4,300 farms as of 2019 and pork is produced mainly for domestic consumption (about 900,000 ton/year) and partially for export (about 2,000 ton/year). Although the export of pork is not a major industry in Japan, since the domestic demand for pork in Japan is increasing in recent years to more than 1.8 million ton/year, the protection of domestic pig industry from CSF and the maintenance of productivity is also a major issue.

The disease is caused by the CSF virus (CSFV), a single-stranded RNA virus of the *Pestivirus* genus of the *Flaviviridae* family. Pigs and wild boars are the virus hosts. Infected animals experience non-specific clinical symptoms due to immunosuppression (1, 2). Clinical forms of the disease vary depending on the virulence of the virus, age of host animals, hygiene management at the farm, and the presence of secondary infections; the clinical forms that have been seen in wild boars are similar to those in pigs (2). CSF can be divided into the following forms: acute, chronic, and persistent. The acute form is characterized by atypical clinical signs such as high fever, anorexia, gastrointestinal symptoms, general weakness, and conjunctivitis. This is followed by neurological signs and skin hemorrhages or cyanosis in different locations of the body 2 to 4 weeks after infection, known as the “typical” CSF signs. Animals with this form usually die 10 to 30 days after CSFV infection. In the chronic form, animals show various non-specific symptoms including fever, listlessness, loss of appetite, decreased growth, and death after 1 month from infection. The persistent form is observed in piglets infected as fetuses through vertical transmission (2). These piglets can become immunotolerant to the virus and can be a constant source of infection. They are able to constantly excrete the virus, even without any clinical symptoms, and are a dangerous virus reservoir until the late onset (2).

CSF outbreaks in pigs have been reported in Central and South America, Europe, Asia, and Africa. In the 1990s, large outbreaks occurred in the Netherlands, Germany, Belgium, and Italy but the disease has now been contained in these Western European countries. These countries are now officially recognized as CSF free, according to the World Organization for Animal Health (OIE) Terrestrial Code (3). Japan suffered from CSF since from the 1880s until the development of a live vaccine using the GPE-strain in the 1960s. The live vaccine was used since 1969, resulting in a sharp decline in the number of outbreaks to zero. The last reported case was recorded in 1992 (4). In 2000, the use of the live vaccine began being restricted before totally ceasing in 2006. Japan was officially recognized as CSF free in 2015, when the OIE began officially recognizing CSF disease status (5). This status was subsequently suspended in September 2018 due to the re-occurrence of CSF in central Japan.

On August 24, 2018, a fallow-to-finish pig farm in Gifu Prefecture, located in the central part of Japan, reported an increase in the number of dying animals to their local veterinary service. At the farm, clinical signs such as fever, loss of appetite, and abortion were more frequently observed prior to the deaths. The farm manager consulted the farm veterinarian, who considered the signs to be caused by heatstroke. On September 9, 2018, CSF viral infection was confirmed by laboratory tests conducted at the National Institute of Animal Health, National Agriculture and Food Research Organization (NIAH-NARO), after an absence of 26 years.

**Figure 1:**
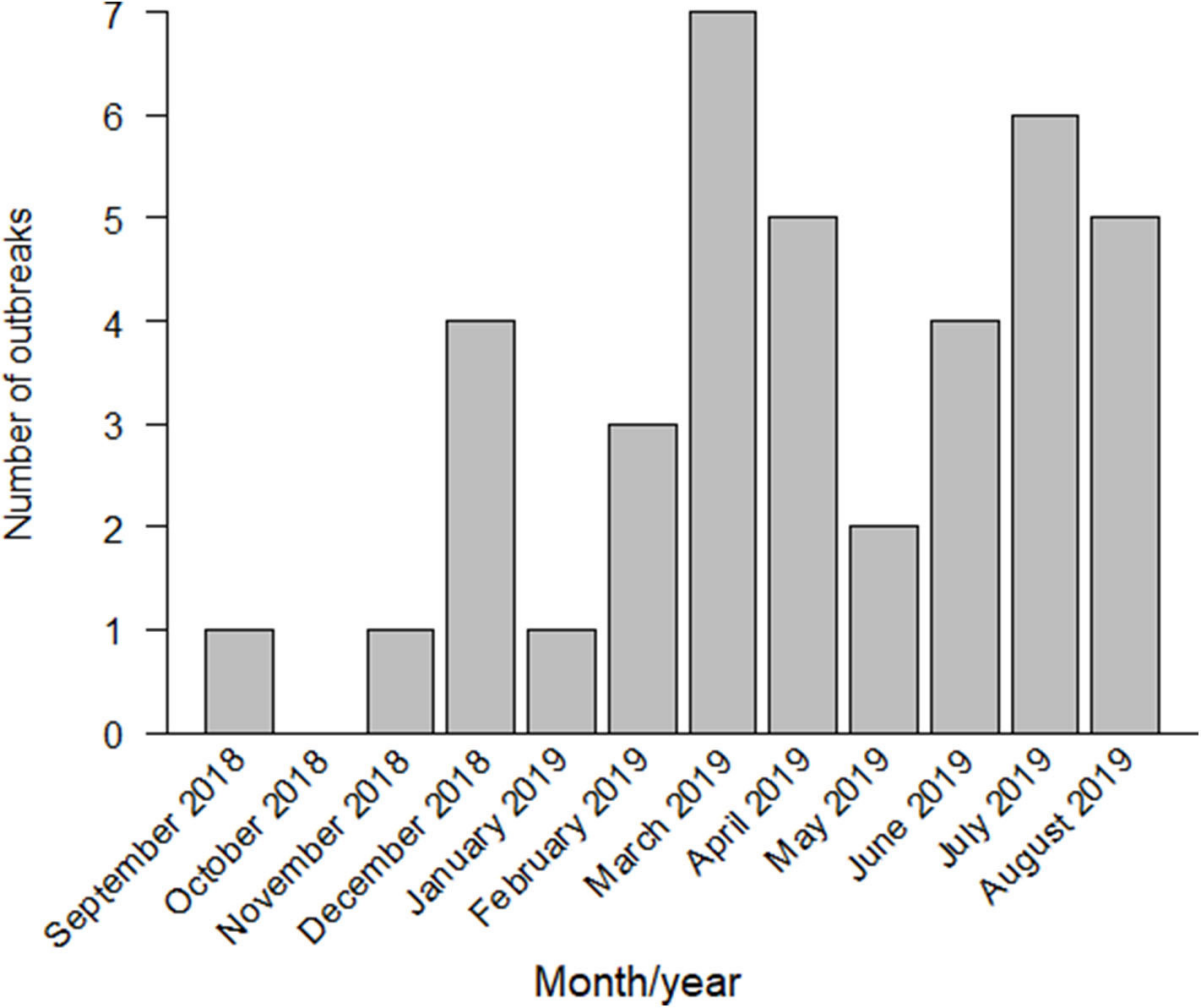
Classical swine fever (CSF) outbreaks reported in Japan from September 2018 to August 2019.

**Figure 2:**
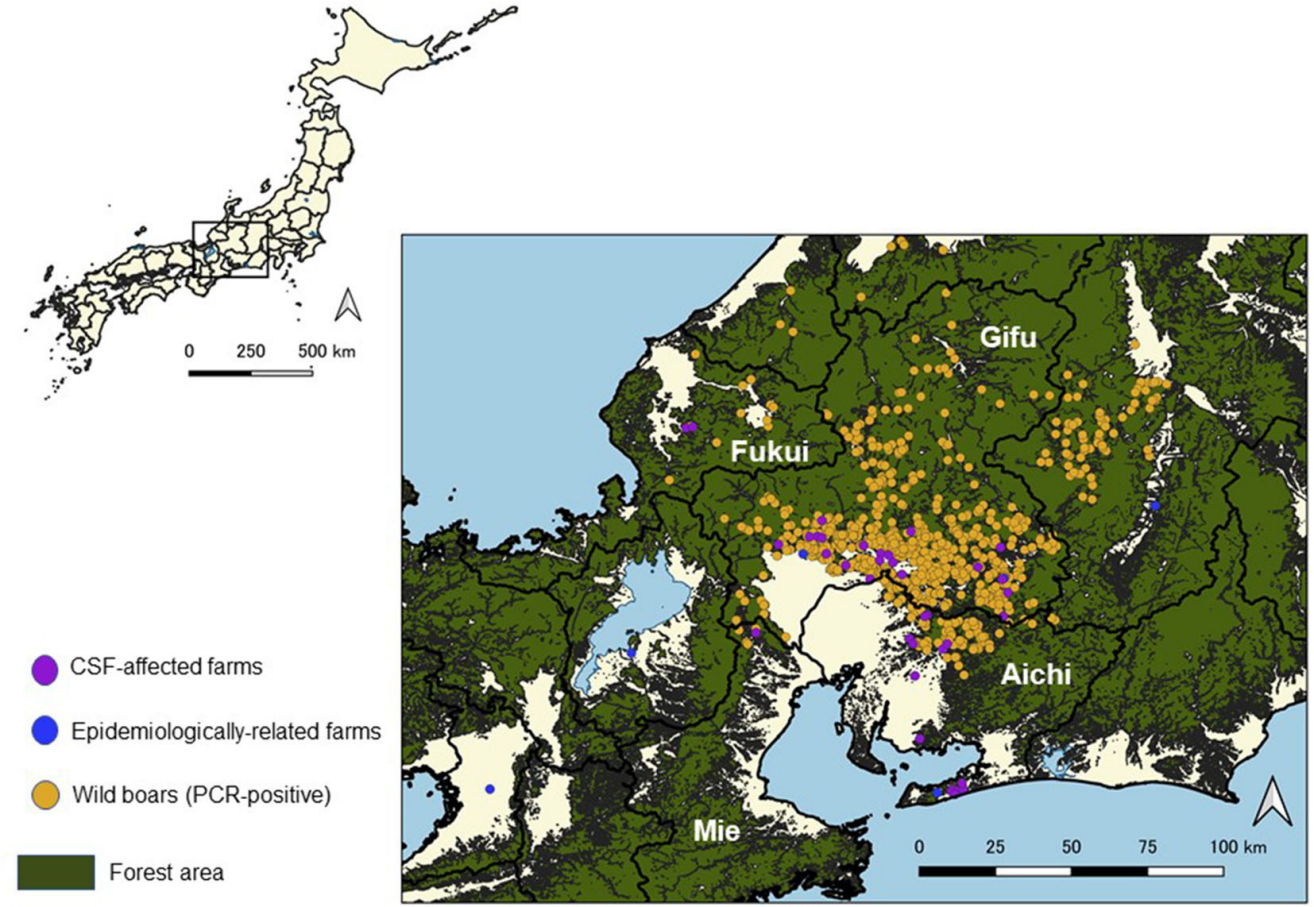
Location of classical swine fever (CSF)-affected farms and virus-positive wild boar cases in Japan from September 2018 to August 2019.

By January 2019, six more outbreaks had been reported near the first infected farm, in the southern area of Gifu Prefecture. In February 2019, the first outbreak in Aichi Prefecture was confirmed in Toyota City, located in the northern area of Aichi. The second outbreak in Aichi Prefecture was reported within the same month, in Tahara City, located in the southern peninsula, almost 47 km away from the infected farm in Toyota City. From March to June 2019, a total of 18 outbreaks had been reported in Gifu and Aichi. In July 2019, the first outbreak in Mie Prefecture and the first outbreak in Fukui Prefecture were reported. By August 2019, 39 outbreaks had been confirmed in Gifu, Aichi, Mie, and Fukui Prefectures (Figures 1, 2).

The virus strain causing the outbreaks in Japan from 2018 to 2020 was found to be the subgenotype 2.1d (6, 7). Subgenotype 2.1d was firstly isolated from the outbreaks in China in 2014 to 2015 (8) and is reported to cause a chronic or moderate form of infection (9–11). It has also been detected in South Korea (12, 13). A phylogenetic study showed that the CSF virus isolated from the 2018 Japan outbreak index case was different from the strains that had caused previous CSF-outbreaks in Japan (6). It has, therefore, been considered that the virus causing the 2018–2020 CSF outbreaks in Japan was newly introduced from the surrounding Asian countries, though the route of introduction or origin of the virus is unclear.

Control measures implemented to contain the CSF outbreaks were based on the Guideline to Control Classical Swine Fever (hereinafter referred to as “the Guideline.” A specific national guideline and the last revised version was published on February 5, 2020 under the Act on Domestic Animal Infectious Disease Control of Japan.) (14). These measures included: stamping-out of all animals in the affected farms, control of movement of animals to a radius of 3 km of the affected farms, control of animal shipment in the area of 3 to 10 km from the affected farms, and disinfection of vehicles at control-points set-up at roads inside the movement control areas. All farms identified as having a relationship with the affected farms were investigated, and all animals confirmed to be infected with CSF virus at these farms were also stamped-out. Killed animals were buried at the affected farms or at burial sites near the affected farms, except for one epidemiologically related farm, whose animals were rendered for incineration due to unavailability of burial sites.

Active surveillance was implemented at farms and in wild boars. Surveillance at farms was implemented within the movement-control areas, within a 3 km radius of the affected farms. Clinical investigation, polymerase chain reaction (PCR), and enzyme-linked immunosorbent assay (ELISA) tests were conducted on blood samples of randomly-selected pigs from the affected farms within 24 h of confirmation of an infection. At least 30 animals were tested per farm to detect a prevalence of 10% with 95% confidence. When a farm had more than two pig houses, at least five animals per pig house were tested even when the total sample size exceeds 30. In these samplings, pigs with clinical symptoms were sampled with priority. The same set of tests was conducted to all farms present within the movement-control area from 17 days after the completion of all control measures at the affected farm. Shipment restrictions in the area of 3 to 10 km from the affected farms were lifted when all the farms within the movement-control area were confirmed to be CSF free by the second round of tests, while movement controls were lifted 28 days after the completion of all control measures on the affected farms.

A nationwide surveillance in wild boars had been implemented since 2006, after the cessation of the use of vaccine in pigs, and after the official recognition of CSF freedom in 2015, 273, and 389 wild boars were tested in 2016 and 2017, respectively with negative results. After the first pig case was confirmed at a pig farm in Gifu Prefecture in September 2018, intensive CSF surveillance in wild boars targeting the area within 10 km of the affected farm started according to the Guideline. As a result of the intensive surveillance, the first positive case of wild boar was found dead within 10 km of the farm with the first pig case. After the first case of wild boar, hunters captured wild boars within a 10 km radius of the sites where infected wild boars were found, in addition to the area around affected farms. The dead or captured wild boars in the surveillance areas were then tested for CSF by local veterinary services. Additionally, all prefectures were requested to test dead wild boars found in their jurisdiction.

The vaccination of wild boars, using bait vaccine, started in March 2019 in Gifu Prefecture and in the adjacent prefectures with CSF-positive wild boars. By October 2019, given that the spread of the disease had not been controlled by improving biosecurity measures at farms, preventive vaccination at pig farms using the live CSF-vaccine began in Gifu and in the eight adjacent prefectures. By the end of December 2019, vaccination expanded to the additional 11 surrounding prefectures.

Descriptive epidemiological analyses provide an overview of the epidemic and shed light on the characteristics of the outbreaks, including the possible factors related to the occurrence of the disease. There are descriptive epidemiological studies on CSF outbreaks that have occurred in the Netherlands, Germany, and Belgium (15–18). These are all good references for countries needing to control the disease.

Regarding the epidemic of CSF caused by subgenotype 2.1d virus strain, the isolation of the virus has been reported (9–13), but the features of outbreaks caused by the specific subgenotype virus strain have not been fully described. As for the re-occurrence of CSF in Japan since 2018, there were studies describing genetic characteristic of the virus (6, 7), pathogenicity in experimental infection (19), and estimating the risk of infection from wild boars (20). However, an overall description of the outbreaks and analyses of clinical manifestations observed during outbreaks have not been reported.

This study is the first report that gives an epidemiological overview of the CSF outbreaks in pig farms by the virus strain of subgenotype 2.1d, which occurred in Japan, for the period from September 2018 to August 2019. The characteristics of the symptoms observed from infected animals and the measures taken at the affected farms described in this study will be a good reference for the countries affected by the epidemic of CSF caused by subgenotype 2.1d, which causes a chronic or moderate form of infection.

## Materials and Methods

### Data Collection

Epidemiological data from the 39 farms where CSF outbreaks occurred in the period from September 2018 to August 2019 were collected using the epidemiological investigation reports. These reports also included information on the preventive measures implemented at the farms. Each epidemiological investigation on an affected farm was conducted by epidemiological investigation team (EIT) from the Ministry of Agriculture, Forestry and Fisheries (MAFF) of Japan. The EIT consisted of veterinary officials of MAFF and veterinary epidemiologists of NIAH-NARO. Most of the investigation activities were implemented on the date or the next date of confirmation of CSF infection and before starting of the stamping-out at the farm. During the epidemiological investigation, managers of affected farms were interviewed and asked about the biosecurity measures implemented at their farms, and about flows of workers and pigs inside and outside of pig houses. Information on structures of affected pig houses and feedstuff was also collected at the on-site investigation. When fences and/or bird-proof nets were installed at affected farms, the way they were installed was checked by the EIT and the EIT confirmed if there were any possibilities of intrusion of wild animals. Brief list of questions used in the EIT investigations are shown in Supplementary Table 1.

Farm locations were extracted from the Domestic Animal Disease Control Map Database of Japan. All farms which did not rear pigs or boars for marketing purposes were classified as non-commercial farms. These included farms being managed by municipalities for breeding or education. Information regarding the number of animals being reared at the affected farms and the duration from infection confirmation to completion of stamping-out was collected from publicly available data from MAFF and prefectural governments.

Data on CSF-positive wild boars, including their location and laboratory test results, were also provided by MAFF.

### Data Analysis

All statistical analyses were conducted using R (R Core Team, 2020). The Fisher's exact test was applied for univariate analyses. CSF-cumulative incidence rates by types of farms and clinical symptoms by types of pigs were compared by applying the test to 2 × 2 contingency tables. The association between the infection in sows and the status of transmission between pig houses was analyzed in the similar method. For the multiple comparisons of CSF-occurrence among farrow-to-finish farms, fattening farms and breeding farms, a 2 × 3 contingency table was prepared and the Fisher's exact test was applied by the “fisher.multcomp” function of the RVAideMemoire package. For the comparison of the number of animals at farms and the number of leucocytes by categories of farms and pigs, the Wilcoxon rank sum test (“wilcox.test” function) was applied.

### Clinical Manifestations at Affected Farms

Information on the clinical manifestation, as observed at disease notification, and the type of pigs showing symptoms (piglets, sows, or fattening pigs) was collected from the following sources: (i) the epidemiological investigation reports completed by EIT (provided by MAFF), (ii) the CSF-EIT meeting reports (published on the MAFF web-site), (iii) the emergency notification reports submitted to the MAFF by the prefectural governments (provided by MAFF), and (iv) the verification reports prepared by Gifu Prefecture on their response to the CSF outbreaks (available on the Gifu prefectural government web-site).

The type of pigs affected, that is, piglets, sows, and fattening pigs, were classified as stated in the above sources. However, some farms only provided data on the age of the affected pigs. In such cases, pigs <3 months (or 90 days) of age were classified as piglets; pigs aged 3 months (or 90 days) and above were classified as fattening pigs, and pigs for breeding purposes were classified as sows. Observed clinical manifestations were classified into eight symptoms; loss of appetite, listlessness, respiratory disorders, fever, cyanosis, diarrhea, death, and neurological symptoms.

The association between the type of pigs (fattening/sows/piglets) and the development of any of the major four symptoms, that is, loss of appetite, listlessness, respiratory disorders, and death, was analyzed for each combination.

### Results of Laboratory Tests of Pigs at Affected Farms

Farms with confirmed CSF infections, following tests conducted after receiving notification, had five or more pigs from each pig house randomly sampled before being stamped-out. The basic sample size was at least 30 animals per farm, to detect a prevalence of 10% with 95% confidence, and as additional conditions to detect the infection more efficiently, at least five animals per pig house, from all the pig houses, with priority in sampling from pigs with clinical symptoms were sampled in accordance with the Guideline. Investigations were conducted to measure the number of leucocytes, and PCR and ELISA tests performed to determine the infection status and spread of the virus at the farm. Blood sampling was conducted by the prefecture's local veterinary service, based on the Guideline, and antigens and antibodies against CSF were tested using PCR and ELISA, respectively. Following infection by CSF virus, the viral antigen is detected in the blood and/or organs of pigs where it grows, before any antibodies can be detected. Accordingly, the status is indicated as PCR(+)/ELISA(–). As the course of infection proceeds, antibodies against the CSF virus can be detected and the status becomes PCR(+)/ELISA(+). After the virus is eliminated from the pigs, the pigs become immune to CSF virus infection and the status is indicated as PCR(–)/ELISA(+). Experimental infections using the virus strain isolated from the cases in Gifu Prefecture indicated that antibodies against the virus are developed on or after 14 days from infection, and that antigen detection lasts for more than 28 days after infection (19).

### Proportion of Dead Animals at Farms

Obligatory daily reporting of the number of dead animals was imposed on farms located within a 3 km radius of an affected farm, a 10 km radius of an infected wild boar, and that had shipped pigs to the common slaughterhouses shared by affected farms, starting from February 2019, following the detection of the 8th case (the first outbreak in Aichi Prefecture). Reports were collected from the affected farms by the local veterinary service who reported the number to the MAFF. The daily proportion of dead animals was calculated by dividing the number of dead animals per day by the total number of animals at the farm on that day. When the total number of animals at the farm on each day was not available, the number of animals at the farm on the date of stamping-out was used as the denominator.

### Data on Geographical Information

Geographical data on administrative divisions (as of 2018) and forested areas (as of 2015) was downloaded from the National Land Numerical Information download service, provided by the Ministry of Land, Infrastructure, Transport and Tourism of Japan, and was used to draw maps. The maps were drawn, and distance measured, by quantum geographic information system (QGIS) version 3.10.

We recorded the distance between the affected farms and the nearest site where a PCR-positive wild boar was found before the farm notified the outbreak. In addition, the shortest distance between affected farms was measured as the distance between an affected farm and the nearest affected farm with a confirmed infection, in which the infection was confirmed before that of the farm in question. This was measured using the distance matrix of the geoprocessing tools of QGIS.

## Results

### Details of the Affected Farms

#### Classification and Comparison by Types of Farms

##### Pig/boar

Out of 39 outbreaks confirmed between September 2018 until August 2019, 38 outbreaks occurred at pig farms and one outbreak at a boar farm. Boars are not common livestock in Japan but there are boar farms where several or a few dozen boars are reared for training hunting dogs or for meat, or often without any particular purpose, as in the affected boar farm. In Gifu Prefecture, 21 out of 44 pig farms and one out of five boar farms were affected. The cumulative incidence rate was 48% (=21/44) for pig farms and 20% (=1/5) for boar farms, and there was no significant difference between the cumulative incidence rate of pig farms and boar farms (*p* > 0.1, Fisher's exact test).

##### Commercial/non-commercial

Five outbreaks occurred at non-commercial farms; four in Gifu Prefecture from November to December 2018, and one in Aichi Prefecture in August 2019. Affected non-commercial farms included: the livestock research institutes of Gifu and Aichi Prefectures, the Gifu Prefectural College of Agriculture, the Gifu Prefectural Park of Livestock, and a boar farm. Thirty-four outbreaks occurred at commercial pig farms. In Gifu Prefecture, 18 out of 41 commercial pig/boar farms were affected and four out of eight non-commercial pig/boar farms were affected. The cumulative incidence rates were, therefore, 44% (=18/41) in commercial farms and 50% (=4/8) in non-commercial farms, with no significant difference between them (*p* > 0.1, Fisher's exact test).

##### Farrow-to-finish/fattening/breeding

The affected 34 commercial pig farms consisted of 28 farrow-to-finish farms, 5 fattening farms, and 1 breeding farm. Two out of five fattening farms were group farms comprising a farrow-to-finish and a breeding farm. In Gifu Prefecture, at commercial pig farms, the cumulative incidence rate of the farrow-to-finish farms was 93% (=13/14 farms), fattening farms was 29% (=4/14 farms), and breeding farms was 8.3% (=1/12 farms) (Table 1). Comparing the rates among the types of farms, the cumulative incidence rate of the farrow-to-finish farms was significantly higher than that of the fattening and breeding farms (*p* < 0.05, multiple comparison).

**Table 1:** Number of classical swine fever (CSF) outbreaks at commercial pig farms in Gifu Prefecture from September 2018 to August 2019, by production type.

**Production type**	**Number of farms**		
	**Affected**	**Not affected**	**Total**
Farrow-to-finish	13	1	14
Fattening	4	10	14
Multiplier	1	11	12
Total	18	22	40

#### Number of Animals

**Figure 3:**
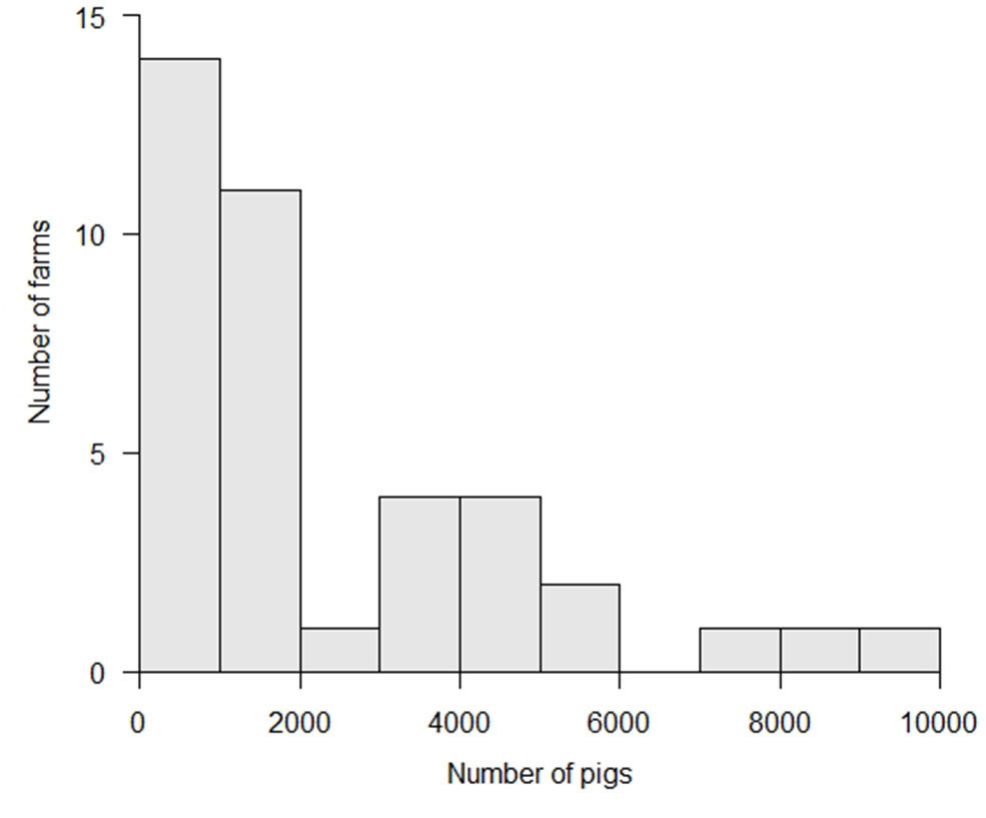
Herd-size (number of pigs) at classic swine fever (CSF)-affected farms.

More than half of the affected farms reared <2,000 animals, with a median number of 1,271 (25–75th percentile: 625–3,622) animals (Figure 3). The median size of these farms was not significantly different from that of all farms in Japan (*p* > 0.1, Wilcoxon rank sum test) (21). For non-commercial farms, the livestock research institutes of Gifu and Aichi Prefectures reared about 500 and 700 animals each, respectively. The other non-commercial farms reared 10 to 20 animals per farm. For commercial farms, the median number of animals reared per farm was 1,556 (25–75th percentile: 976–4,007) animals, ranging between 250 and 10,000 animals.

In Gifu Prefecture, 22 out of 49 pig/boar farms were affected and 27 farms were not affected. The median number of animals reared at the 22 affected farms was 1,277 (25–75th percentile: 594–2,916) animals, while the median number of animals reared at the 27 non-affected farms was 519 (25–75th percentile: 127–1,584) animals. Therefore, the number of animals reared at affected farms was significantly higher (*p* < 0.05, Wilcoxon rank sum test).

#### Number of Days From Diagnosis to Completion of Stamping-Out

**Figure 4:**
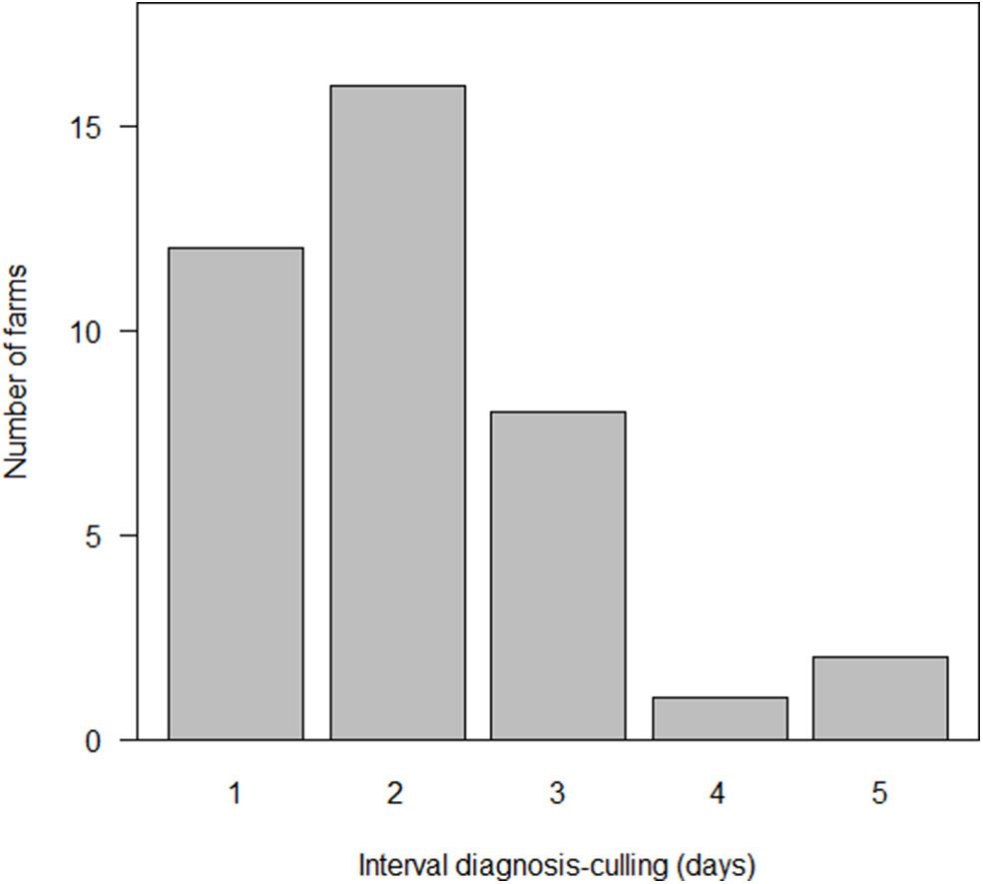
Distribution of the interval between laboratory confirmed diagnosis and the completion of culling at 39 farms affected by classical swine fever (CSF) in Japan, 2018–2019.

The time between definitive diagnosis and the completion of stamping-out ranged between 1 and 5 days, with a median of 2 days (25–75th percentile: 1–3 days) (Figure 4). There were 11/39 farms that required 3 days or more to complete stamping-out, of which 10 of these had either more than 4,000 animals or constituted a pig farm complex, resulting in multiple farms needing to be slaughtered simultaneously. The median number of animals at these 11 farms was 4,189 (25–75th percentile: 3,520–5,215) animals and was significantly larger than that of the other 28 farms (*p* < 0.01, Wilcoxon rank sum test).

### Clinical Manifestations and Transmission of Virus Within Farms

#### Common Clinical Manifestations

##### Fever

Eleven of the 38 pig farms suspected CSF by fever and made notification. After including results from the on-site inspections following notification, pigs with a fever over 40°C were observed at 30/38 pig farms.

##### Decrease in the number of leucocytes

Pigs from all affected farms were noted to have a decreased leucocyte count to <10,000 cells/μl. Leucocyte counts were not measured at the wild boar farm because of their aggressiveness. The leucocyte level was measured on 939 CSF-positive pigs [PCR(+) and/or ELISA(+)] and 3,005 CSF-negative pigs [PCR(–) and ELISA(–)]. The median leucocyte level within CSF-positive pigs was 8,490 (25–75th percentile: 5,900–13,000) cells/μl, which was significantly lower than that of CSF-negative pigs (*p* < 0.01, Wilcoxon rank sum test).

#### Clinical Manifestations Leading to the Notification and the Results of Laboratory Tests

Clinical manifestations of fattening pigs were the reason for notification at 18 out of 34 commercial farms. Among these 18 farms, death was the common reason for notification in 12 farms.

In 9 out of 34 commercial farms, clinical manifestations of sows led to notification. At all nine farms, the reason for notification was loss of appetite, with death also cited as a reason in one of these nine farms.

In 6 out of 34 commercial farms, clinical manifestations in piglets led to notification. The death of weaning pigs was the reason for notification in four out of six farms while loss of appetite in sows was also cited as a reason in one of the four farms. As for the other two farms, fever and listlessness of weaning pigs were the reasons for notification at one farm, and listlessness of piglets as well as loss of appetite and listlessness of sows were the reasons in the other.

Table 2 shows the observed clinical manifestations and the results of laboratory tests according to farm level.

**Table 2:** Reported clinical signs by types of pigs and the results of serological tests on CSF at the 34 affected commercial farms.

**Type of pigs**	**Reported clinical manifestation**	**No. of farms with clinical signs**	**Results of serological tests on CSF at farm level**^**a**^		
			**PCR-positive/ELISA-negative**	**PCR-positive/ELISA-positive**	**PCR-negative/ELISA-positive**
**Fattening pig**		18	1	6	11
	Death	12	1	3	8
	Fever	7	0	3	4
	Listlessness	8	0	4	4
	Loss of appetite	7	0	3	4
	Respiratory disorders	5	0	0	5
	Diarrhea	2	0	1	1
	Cyanosis	2	0	0	2
	Decreased growth	1	0	0	1
**Sow**		9	2	3	4
	Loss of appetite	9	2	3	4
	Fever	4	2	1	1
	Listlessness	2	0	1	1
	Death	1	1	0	0
**Piglet**		6	1	3	2
	Death	4	1	2	1
	Listlessness	3	0	2	1
	Cyanosis	2	1	0	1
	Fever	2	0	2	0
	Loss of appetite	1	0	1	0
	Neurological symptom	1	0	1	0
	Diarrhea	1	0	0	1

a Classification of farms by the results of serological tests. PCR-positive/ELISA-negative, all animals tested at the reporting and before culling were PCR-positive but without CSF-virus-specific antibodies; PCR-positive/ELISA-positive, at least one animal tested at the reporting or before culling was PCR-positive and with CSF-virus-specific antibodies; PCR-negative/ELISA-positive, at least one animal tested at the time of reporting or before culling was with CSF-virus-specific antibodies but PCR-negative.

*PCR, polymerase chain reaction; ELISA, enzyme-linked immunosorbent assay*.

Other than the farms that notified CSF by clinical manifestations, in 4 out of 34 commercial farms, the clinical manifestations were not observed by farm managers, where the infection was detected by PCR and ELISA tests applied as a part of the movement/shipment-control areas. After the detection by the laboratory tests, pigs with fever were confirmed at two farms, but pigs at the other two farms remained asymptomatic. Pigs tested ELISA(+) at two out of four farms, PCR(+)/ELISA(+) at one farm without fever, and PCR(–)/ELISA(+) at one farm with fever. In the other two farms, pigs only tested PCR(+)/ELISA(–) but one of the farms had pigs with fever.

As a result of analyses on the association between the type of pigs and the development of symptoms, it was indicated that respiratory disorders and loss of appetite significantly led to notification more frequently in fattening pigs and in sows, respectively (*p* < 0.05 and *p* < 0.01, Fisher's exact test). Death was significantly less frequently cited as a reason for notification in sows (*p* < 0.05, Fisher's exact test).

#### Abnormal Birth

Abnormal births, including abortion and stillbirth, were reported only by three farms during the epidemiological investigation interviews, while stillbirths were recorded in the daily reports from 10 other farms traced up to 60 days before confirmation of infection. In 9 out of these 13 farms, the pig houses with recorded stillbirths were confirmed to be affected with CSF afterwards. However, none of these farms suspected that the abnormal births were a clinical manifestation of CSF infection.

#### Death and Proportion of Dead Animals

Based on the records of the daily number of dead animals at the farms traced up to a maximum of 60 days before infection was confirmed, 30 out of 39 affected farms had observed their animals dying before notification. In 12 out of 30 farms, the cause of the death was considered to be due to weakness or being crushed (in suckling pigs), diarrhea (in weaning pigs), streptococcus's infection, pneumonia, gastroenteritis, and stress or growth insufficiency (in weaning and fattening pigs). CSF was not suspected as the cause according to both the local veterinary service and the supervising veterinarian since the other pigs being reared in the same pens or pig houses as the dead pigs did not have any abnormal symptoms. In the other 18 farms, CSF was suspected, and the death led to notification.

**Figure 5:**
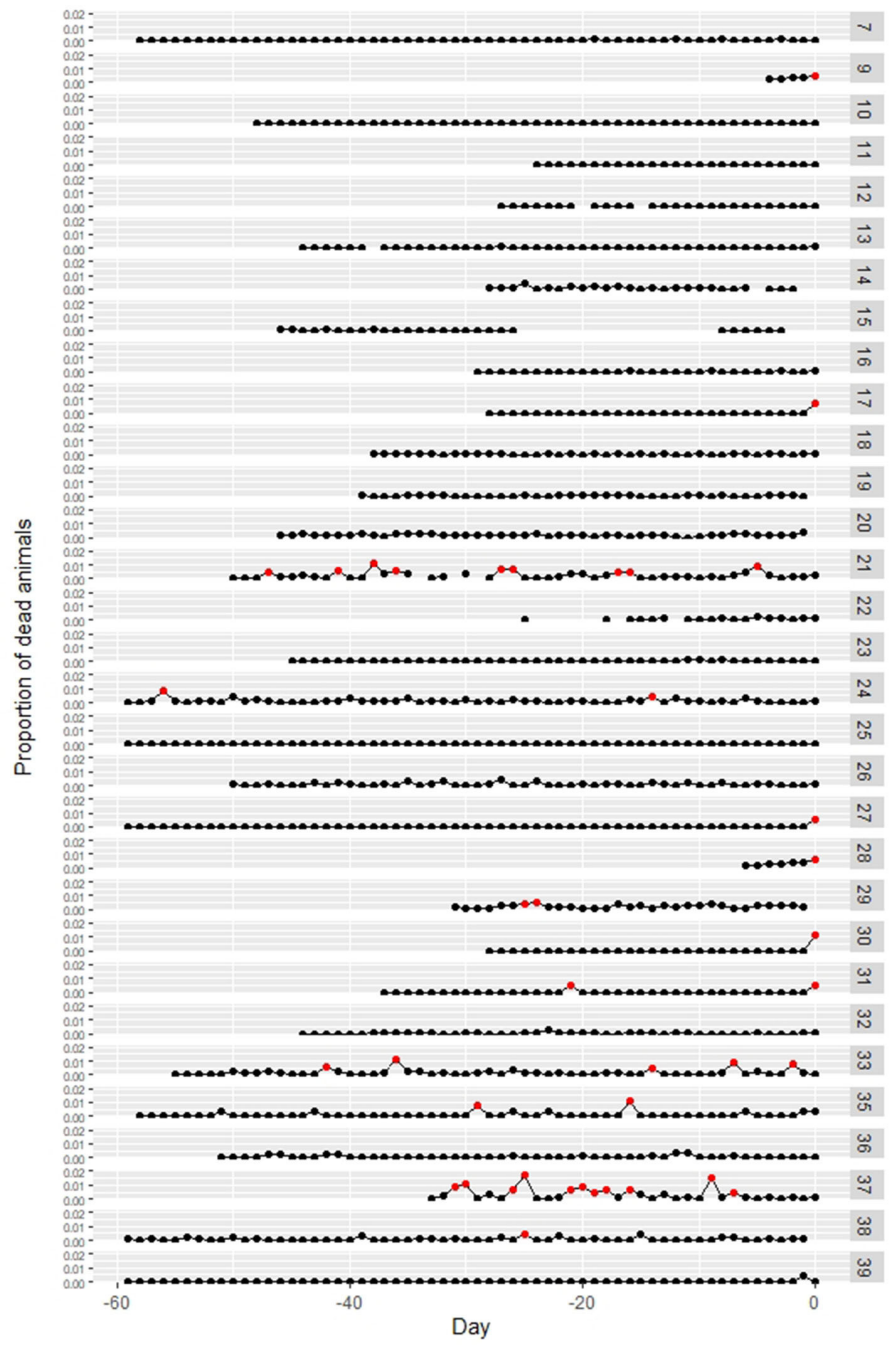
Changes in the proportion of dead animals at classical swine fever (CSF)-affected farms. Numbers at the right side indicate case numbers. The proportion over 0.5% is marked as red point. Day 0 = report date. Data were not available for Case no. 1–6, 8, and 34.

Data on the daily number of dead animals before notification were available on 31 of the affected farms. In 6 out of the 31 farms, the proportion of dead animals on the date of notification was more than 0.5% (Case no. 9, 17, 27, 28, 30, 31). Three of these six farms notified due to the increase in the number of dead weaning pigs, and the other three farms due to the death of their fattening pigs. Other than those six farms, there was no observed increased number of dead animals from the other farms on the date of notification (Figure 5). For the other 25 farms, temporary increases in the proportion of dead animals to more than 0.5% were observed before the dates of notification in six farms (Case no. 21, 24, 29, 33, 35, 37), but the cause of the increase was crushing death in suckling pigs or abortion.

#### Transmission Between Pig Houses by Pig Flow (Intra Farm Pig Movement)

In the 34 commercial farms, including the two farms without any clinical manifestations, infection was limited to one pig house in eight farms and confirmed in all the pig houses in the other eight farms. In the other 18 farms, infection was confirmed in more than two, but not all, pig houses. Transmission of the infection between pig houses by pig flow was assumed to have occurred when there was a record of the infected pigs having moved between pig houses, and when both pig houses were confirmed to be affected. Based on the epidemiological investigations and movement records of the infected pigs, transmission by pig flow was strongly suspected in 7 of the 26 farms, with infection confirmed in multiple pig houses. In five of the farms, the viral spread by pig flow was refuted since the farms were fattening farms and/or the pigs had not moved between pig houses. In the other 14 farms, the transmission routes between pig houses remained unclear because either there were no records of the movement of pigs between pig houses or because almost all of the pig houses in the farm had been found to be infected with no trace as to the source of the infection within the farm.

#### Transmission Between Stages of Pigs

The infection was not limited to a single stage in 23 out of 29 commercial farms rearing multiple stages of pigs (28 farrow-to-finish farms and one breeding farm). In one the other six farms, infection was confirmed only in fattening pigs (five of the six farms) and in sows (one of the six farms).

The association between the infection in sows and the status of transmission between pig houses is shown in Table 3. Infection in multiple pig houses was observed more frequently when there was infection of sows (*p* < 0.01, Fisher's exact test).

**Table 3:** CSF-infection among sows and the spread of classical swine fever (CSF) viruses between pig houses.

**Infection in sows**	**Number of CSF-affected farms rearing multiple stages of pigs**		
	**Spread of CSF viruses in each farm**		**Total**
	**Limited to a single pig house**	**Observed in multiple pig houses**	
Yes	2	19	21
No	5	3	8
Total	7	22	29

### Surrounding Environment of the Affected Farms

#### Distribution of Infected Wild Boars

In Gifu Prefecture, wild boars confirmed as PCR-positive were frequently detected near the affected farms. On the other hand, PCR-positive wild boars were not found near the affected farms located at the southern peninsula of the Aichi Prefecture (Figure 2).

Twenty-eight out of 38 affected farms, excluding the first affected farm, were located within 5 km from PCR-positive wild boars detected before the notification of an outbreak at each farm. Out of the 28 farms, 23 farms were located in the southern area of Gifu Prefecture or in the adjacent northern area of Aichi Prefecture, two farms were located in the central part of Aichi Prefecture, and the other three farms were located in Mie and Fukui Prefectures.

#### Distance Between Affected Farms

The median distance between affected farms and the nearest other affected farm was 6.95 km (25–75th percentile: 2.35–11.38 km). Four farms were located within a distance of 1 km, with one of these four farms was located in a pig farm complex in the southern peninsula of Aichi Prefecture. The other three farms were located in the northern area of Aichi Prefecture, with two of the three farms adjoining each other and the nearest affected farm being the one in common. One of these farms was an affiliated farm of one of the other two farms.

### Livestock Health Management at Affected Farms

#### Feed

In 33 out of 39 farms, only commercial feed was used. The other six farms used feed other than commercial feed (Table 4), with one of these six farms being the boar farm which used rice bran, wasted rice, breadcrumbs discarded from food factories, and vegetable scraps discarded by neighboring farmers. The other five farms using non-commercial feed were commercial pig farms using confectionery residues such as biscuit crumbs (two farms), breadcrumbs (one farm), weeds around the farm (one farm), and liquid feed made with food wastes including table leftovers and cooking residues (one farm) as feed.

**Table 4:** General characteristics of the 39 farms affected by classical swine fever.

	**Number of farms**
**Structure of affected pig houses**	
Windowless	0
Semi-windowless^a^	18
With open windows^b^	21
**Feedstuff**	
Commercial feed only	33
Other than commercial feed	6

a One of the 18 semi-windowless pig-houses had open-air paddocks.

b *Two of the 21 pig-houses with open windows had open-air paddocks*.

#### Measures to Prevent Intrusion of Wild Boars

Fences around farms were installed at 26 of the 39 farms, but only 14 farms had complete fences protecting them against intrusion by wild boars. In addition, 15 of the 39 farms had installed electric fences, but the fences were complete at only 12 farms (Table 5).

**Table 5:** Preventive measures implemented at the 39 farms affected by classical swine fever.

	**No. of farms**	**Proportion (*N* = 39)**
**1. Measures at the boundaries of the farms**		
Installation of fences without electricity^a^	26	67%
- part of fences were left open and/or gaps or damages present	12	31%
- without any defects	14	36%
Installation of electric fences^a^	15	38%
- parts of fences were left open and/or gaps or damages present	3	8%
- without any defects	12	31%
Covering ground with hydrated lime at entry points	15	38%
Disinfection of vehicles at entry points	28	72%
- by power sprayer^b^	25	64%
- by disinfection baths^b^	6	15%
- by portable sprayer^b^	2	5%
- by disinfection mats^b^	2	5%
Changing footwear of persons entering the farms	29	74%
Changing clothes of persons entering the farms	23	59%
**2. Measures to prevent intrusion into pig houses**		
Covering windows of pig houses with bird-proof nets	25	64%
- gaps or damage present	10	26%
- without any defects	15	38%
Changing boots at the entrances of each pig house	22	56%
Changing gloves and clothes at the entrances of each pig house	6	15%

a Fourteen farms had some electric fences and some fences without electricity; 12 farms had fences without electricity only; one farm had electric fences only; and 12 farms did not have any fences.

b *Farms were applying one or more of the ways to disinfect vehicles at entry points*.

#### Biosecurity Measures at Farm Boundaries

Disinfection of vehicles, such as feed transporters, at farm boundaries was implemented at 28 of the 39 farms. At 5 of the remaining 11 farms, the ground surface at the entrance of the farms was covered with lime. To prevent farm workers from bringing in the virus, changing of boots and clothes at the farm entrances were implemented in more than half of the affected farms (Table 5).

#### Biosecurity Measures at the Border of Pig Houses

As for the structure of the pig houses, there were no windows or filters installed on the openings of the pig houses at 21 of the 39 farms. Openings were covered with curtains at the other 18 farms. There were three farms with open-air paddocks, one of which was a commercial farm (Table 4). To prevent entry of the virus into the pig houses, more than half of the affected farms installed bird-proof netting on the openings of the pig houses, but some of them had gaps or breakages and complete netting was installed at only 15 farms. Changing of boots at each entrance of the pig houses was implemented at more than half of the affected farms (Table 5).

## Discussion

### Details of the Affected Farms

Regarding the type of management, 80% of the affected commercial farms were farrow-to-finish farms. In Gifu Prefecture, the incidence at farrow-to-finish farms was significantly higher than in other farms. This may be due to the fact that in a farrow-to-finish farm, the management of both piglet production and shipment of fattening pigs requires frequent movements of pigs within the farm, with frequent handling, which increases the risk of introducing the virus into the farm. In a case-control study of the outbreaks of foot-and-mouth disease (FMD) in Japan, it was indicated that the risk of infection was higher in farrow-to-finish farms than in fattening farms (22). The reason is considered that sows and piglets in farrow-to-finish farms require more frequent care than pigs in fattening farms and that the disease transmission via direct contact with animals and contaminated fomites tends to occur more frequently in farrow-to-finish farms. Other studies on African swine fever (ASF) outbreaks in Estonia (23) and CSF in the Netherlands (24) have also indicated that the incidence tended to be higher in farms rearing sows with piglets and fattening pigs.

The number of animals at affected farms was significantly larger when comparing affected and non-affected farms in Gifu Prefecture. At large farms, the infection risk may be higher when the number of sows is larger, leading to a larger number of employees engaged in the management of breeding and feeding, as well as the larger number of people entering and leaving the farm for purposes such as transporting feed and shipping fattening pigs. These conditions have not been investigated at non-affected farms, therefore, case-control studies would be necessary for further analysis. On the CSF outbreaks in the Netherlands, a case-control study (25) and a survival analysis (24) reported that the risk of CSF infection was higher on farms with more than 500 animals.

In the CSF outbreak reported in this paper, the median number of days from diagnosis to complete stamping-out was 2 days, including farms with ~10,000 animals. Minimizing the period between infection and stamping-out is important in order to prevent the spread of diseases. In 2010, the spread of FMD in Japan was worsened by delays in stamping-out (22), and due to this fact, the guidelines for specific animal diseases including CSF was revised to include the time limit for the containment measures. The Guideline stipulates that stamping-out should be completed within 24 h, for farms with 1,000 to 2,000 heads of fattening pigs, and that carcasses should be buried or burned within 72 h after confirmation of infection. On the CSF outbreaks in the Netherlands between 1997 and 1998, the median size of the affected farms was 1296.5 animals (25–75th percentile: 800–1,800 animals), and it was reported that 70% of the affected animals were killed within 1 day (26, 27). The relatively longer days required in the affected farms in Japan would reflect relatively large number of animals (median size was 1,271 and 25 and 75th percentile: 625–3,622) reared at infected farms and caused shortage in the available human resources. To complement the shortage of human resources, MAFF coordinated mobilization of official veterinarians of MAFF and surrounding prefectural governments to the affected farms and for several large farms, and the Self-Defense Forces were also deployed to assist activities related to the containment measures. For example, in Gifu Prefecture, a median number of 1,670 (25–75th percentile: 1,108–4,321) people engaged in control activities at an affected farm and the Self-Defense Forces were deployed at 6 out of 20 outbreaks, in which 1,662 to 9,858 animals were subject to stamping-out.

### Clinical Manifestations and Transmission of Virus Within Farms

In the recent outbreaks of CSF in Japan, fever and leukopenia were observed in many cases. In the infection experiment of Japanese isolates, fever over 40°C and leukopenia (<10,000 cells/μl) were observed before the fever had started (19). Fever and leukopenia have been reported as common symptoms of CSF infection in the experiments of other strains (1, 2, 26, 28). Non-specific symptoms such as fever and loss of appetite are common for many diseases and frequently observed at farms, hence they are unlikely to lead to notification. Previous reports have also pointed out that there are cases where notification is delayed due to these symptoms being misdiagnosed as other diseases with clinical symptoms not detected until secondary infection occurs (1, 2, 15, 26).

The results of laboratory tests on the affected farms indicate that the infection in fattening pigs tended to take time to develop clinical manifestations, leading to late notification. Fattening pigs are kept in groups with continuous feeding, therefore, it might be difficult to recognize abnormalities when their appetite is low in the early stages of infection. Notifications may only be made after the appearance of dead pigs, after the number of infected pigs has increased. In addition, there were significantly more cases of respiratory symptoms leading to notifications among fattening pigs than among sows and piglets. It is also possible that CSF infections worsen the clinical symptoms of fattening pigs which have already been infected with other respiratory diseases.

The piglet deaths at the time of notifications were observed in the weaning pigs of each farm. In CSF, piglets suffering from vertical infection are known to be persistently infected and it is possible that these piglets only developed clinical symptoms after weaning. For the piglets showing PCR(+)/ELISA(+), it is thought that viruses are not eliminated despite antibody production. The same condition has been reported in infection experiments with low to moderately pathogenic strains of CSF (29, 30).

In most cases, increases in the number of dead animals to more than 0.5% of the total number was not observed. This result was concordant with the result of the study of experimental infection using the virus strain isolated from the 2018 Japan outbreak. In that study, no infected animals died during the study period up to 28 days post-infection (19). These data suggest that detection of CSF infections by death is difficult in sub-acute CSF infections because the proportion of animals dying does not change significantly.

About 80% of the farms (23/29 farms) had infection in pigs at multiple stages. Infections confined to a single stage were observed mainly in the farms housing only fattening pigs. In addition, this study showed the association between the infection in sows and the occurrence of infection in multiple pig houses. It is also suggested by a previous study using a simulation model that the on-farm infection started in sows could only be noticed clinically when transmitted to weaning or fattening pig groups (31). These studies suggest that infection in sows would cause infection in piglets. These piglets move to other pig houses and become a source of infection to other pig houses and finally detected when they show clinical signs. It is important to note that transmission through the movement of infected pigs cannot be prevented by strengthening biosecurity measures such as disinfection.

### Source of Infection to the Farms

The geographical distribution of outbreaks and infected wild boars in Gifu Prefecture suggested that infected wild boars around the farm might have been the source of infection. The fact that many of the outbreak farms were within 5 km of the proximate infected wild boar detection sites suggested that infected wild boars were the source of infection to the farms. A study on the geographic analysis of the risk of infection from infected boars for the recent Japanese CSF outbreak indicated that the risk was dependent on the distance to the infected boar and that the risk of infection extended to farms within 5 km (20). However, among the farms located within 5 km of the proximate infected wild boar, the intrusion of wild boars into farms was not confirmed by witnessing any signs or footprints of food exploring on livestock, except for one outbreak at the Gifu Prefectural Park. This could indicate that the virus carried by wild boars in the area surrounding the farms might have been secondarily carried into the farms by other wild animals or persons. It is also possible that small wild animals, such as rats and wild birds including crows, could also carry the virus into the farms, although it has not been proven that these animals can transmit the virus to date, and further verification is needed (25, 32, 33).

To enhance the biosecurity measures conducted at pig farms, MAFF has provided several rounds of guidance since the first outbreak in September 2018 on how to comply with the biosecurity standards at farms, including countermeasures against the entry of wild animals. Although vaccination for pigs began in prefectures with infected wild boars in October 2019, biosecurity measures at farms will continue to be important.

As for the outbreaks at the southern peninsula of Aichi Prefecture, infected wild boars were not found near the affected farms. Therefore, the involvement of infected wild boars on those outbreaks is unclear. Genetic analysis has shown that the there is a relationship between strains isolated from infected farms in an area with infected wild boars in Gifu Prefecture and strains isolated from some of the outbreak farms in the southern peninsula area of Aichi Prefecture. Since there was no epidemiological relationship found between those farms, it is considered that the long-distance transmission may have occurred indirectly through vehicles or other fomites traveling between these areas (34).

Considering the role of wild boars in the spread of the disease, the surveillance in wild boars is an important issue. At present in Japan, there are difficulties in conducting and continuing the surveillance of wild boars to monitor CSF infection mainly because of the lack of specific legal and organizational system for disease surveillance in wild animals. Future control plans on CSF in Japan should be discussed with more detailed investigation on the interaction between the infection in pigs and that in wild boars.

If the distance between farms is <1 km, there is a possibility of occurrence of local transmission (35). As for the affected farms, the farms possibly affected by local transmission were located in two areas in Aichi Prefecture, that is, one in the southern peninsula area and one in the northern area adjacent to Gifu Prefecture. Full genome analysis showed that the virus strains isolated from two of the three farms in the northern area were closely related to each other, but the remaining one was different. The virus from this one farm and the virus isolated from five farms in the southern peninsula area were closely related (34). This suggests that in some cases, transmission of the virus was considered to be occurring between neighboring farms. In contrast, more farms were located more than 1 km apart from each other, suggesting that in many farms, outbreaks were caused by factors other than local transmission such as transmission through people, fomites, or wildlife that had some contact with infected wild boars.

For the previous CSF outbreaks, feedstuffs such as kitchen residues were considered to be one of the major sources of infection on farms (15). It is unlikely that swill feeding was the cause of the outbreaks during the 2018 Japan outbreak as only one of the six farms using non-commercial feeds was feeding kitchen waste residues. The other four farms using non-commercial feeds mainly used food plant residues which did not include meat. Therefore, there was no possibility of contamination by the meat. For the two farms that also fed vegetable scraps and weeds, the possibility that these feeds were the source of infection cannot be ruled out, as infected wild boars have been found in the areas surrounding the vegetable scrap and weed collections.

This study mainly focuses on the features of the affected farms and the comparison between affected farms and non-affected farms have not been conducted. To elucidate the factors influencing the risk of CSF infection, comparison between infected and non-infected farms will be necessary. Whether there is a difference in the risk of occurrence due to the structure of pig houses or other status of biosecurity measures will need to be verified through case-control studies and other studies in the future.

## Conclusion

It appears that the current CSF outbreaks in Japan were caused by a virus originating from neighboring countries that spread to pig farms, but the specific route of entry into Japan is unknown. Since most of the infections have occurred in the areas where infected wild boars have been detected and the areas with infected farms are expanding with the expansion of the range of infected wild boars, it is likely that infected wild boars are the main source of infection. Clinical symptoms are non-specific and are difficult to detect during the early stages of the infection. In areas at high risk of infection, daily clinical observation and early testing for pigs showing loss of appetite and listlessness are required. Areas with infected wild boars are still expanding more than a year after the first outbreak. It is necessary to continue to strengthen biosecurity measures at farms located in areas with infected wild boars and to monitor the distribution of infected wild boars.

## Data Availability Statement

The raw data supporting the conclusions of this article will be made available by the authors, without undue reservation.

## Author Contributions

YH conceived the study. YS and TY analyzed the data and wrote the main manuscript text. YH, YM, KS, and EY contributed to the interpretation of the results and helped draft the manuscript. All authors reviewed the manuscript.

## Conflict of Interest

The authors declare that the research was conducted in the absence of any commercial or financial relationships that could be construed as a potential conflict of interest.

## References

[1] Moennig V Floegel-Niesmann G Greiser-Wilke I. Clinical signs and epidemiology of classical swine fever: a review of new knowledge. Vet J. (2003) 165:11–20. 10.1016/S1090-0233(02)00112-0 12618065

[2] Blome S Staubach C Henke J Carlson J Beer M. Classical swine fever—an updated review. Viruses. (2017) 9:1–24. 10.3390/v9040086 28430168 PMC5408692

[3] OIE Recognition of the Classical Swine Fever Status. (2019). Available online at: https://www.oie.int/fileadmin/Home/eng/Animal_Health_in_the_World/docs/pdf/Resolutions/2019/A_R22_CSF_status.pdf (accessed May 7, 2020).

[4] MAFF Annual Statistics of Domestic Animal Infectious Diseases. (1937–2019) (2019). Available online at: https://www.maff.go.jp/j/syouan/douei/kansi_densen/attach/pdf/kansi_densen-162.pdf (accessed May 7, 2020).

[5] OIE Resolutions adopted by the World Assembly of the OIE Delegates during their 83rd General Session. (2015). Available online at: https://www.oie.int/fileadmin/Home/eng/About_us/docs/pdf/Session/A_RESO_2015_public.pdf (accessed May 7, 2020).

[6] Postel A Nishi T Kameyama KI Meyer D Suckstorff O Fukai K Becher P. Reemergence of classical swine fever, Japan, 2018. Emerg Infect Dis. (2019) 25:1228–31. 10.3201/eid2506.181578 30870139 PMC6537743

[7] Nishi T Kameyama K Kato T Fukai K. Genome sequence of a classical swine fever virus of subgenotype 2.1, isolated from a pig in Japan in 2018. Am Soc Microbiol. (2019) 8:e01362–18. 10.1128/MRA.01362-18 30687824 PMC6346156

[8] Leng C Zhang H Kan Y Yao L Li M Zhai H. Characterisation of newly emerged isolates of classical swine fever virus in China, 2014–2015. J Vet Res. (2017) 61:1–9. 10.1515/jvetres-2017-0001 29978049 PMC5894411

[9] Luo Y Ji S Liu Y Lei JL Xia SL Wang Y. Isolation and characterization of a moderately virulent classical swine fever virus emerging in china. Transbound Emerg Dis. (2017) 64:1848–57. 10.1111/tbed.12581 27658930

[10] Zhang H Leng C Tian Z Liu C Chen J Bai Y. Complete genomic characteristics and pathogenic analysis of the newly emerged classical swine fever virus in China. BMC Vet Res. (2018) 14:204. 10.1186/s12917-018-1504-2 29940930 PMC6019732

[11] Zhou B. Classical swine fever in China—an update minireview. Front Vet Sci. (2019) 6:187. 10.3389/fvets.2019.00187 31249837 PMC6584753

[12] An DJ Lim SI Choe SE Kim KS Cha RM Cho IS. Evolutionary dynamics of classical swine fever virus in South Korea: 1987–2017. Vet Microbiol. (2018) 225:79–88. 10.1016/j.vetmic.2018.09.020 30322538

[13] Choe S Cha RM Yu D-S Kim K-S Song S Choi S-H. Rapid spread of classical swine fever virus among South Korean wild boars in areas near the border with North Korea. Pathogens. (2020) 9:244. 10.3390/pathogens9040244 32218239 PMC7238106

[14] MAFF Guideline to Control Classical Swine Fever. (2020). Available online at: https://www.maff.go.jp/j/syouan/douei/katiku_yobo/k_bousi/attach/pdf/index-25.pdf (accessed May 20, 2020).

[15] Fritzemeier J Teuffert J Greiser-Wilke I Staubach C Schlüter H Moennig V. Epidemiology of classical swine fever in Germany in the 1990s. Vet Microbiol. (2000) 77:29–41. 10.1016/S0378-1135(00)00254-6 11042398

[16] Mintiens K Deluyker H Laevens H Koenen F Dewulf J De Kruif A. Descriptive epidemiology of a classical swine fever outbreak in the Limburg Province of Belgium in 1997. J Vet Med Ser B. (2001) 48:143–9. 10.1046/j.1439-0450.2001.00429.x 11315525

[17] Stegeman A Elbers A De Smit H Moser H Smak J Pluimers F. The 1997–1998 epidemic of classical swine fever in the Netherlands. Vet Microbiol. (2000) 73:183–96. 10.1016/S0378-1135(00)00144-9 10785327

[18] Pluimers FH De Leeuw PW Smak JA Elbers ARW Stegeman JA. Classical swine fever in The Netherlands 1997–1998: a description of organisation and measures to eradicate the disease. Prev Vet Med. (1999) 42:139–55. 10.1016/S0167-5877(99)00085-9 10619153

[19] Kameyama KI Nishi T Yamada M Masujin K Morioka K Kokuho T Fukai K. Experimental infection of pigs with a classical swine fever virus isolated in Japan for the first time in 26 years. J Vet Med Sci. (2019) 81:1277–84. 10.1292/jvms.19-0133 31292349 PMC6785620

[20] Hayama Y Shimizu Y Murato Y Sawai K Yamamoto T. Estimation of infection risk on pig farms in infected wild boar areas—epidemiological analysis for the reemergence of classical swine fever in Japan in 2018. Prev Vet Med. (2020) 175:104873. 10.1016/j.prevetmed.2019.104873 31896501

[21] MAFF Census of Libestock Industry 2019. (2019). Available online at: https://www.e-stat.go.jp/stat-search/files?page=1&layout=datalist&toukei=00500222&tstat=000001015614&cycle=7&year=20190&month=0&tclass1=000001020206&tclass2=000001134566&stat_infid=000031878373 (accessed May 20, 2020).

[22] Muroga N Kobayashi S Nishida T Hayama Y Kawano T Yamamoto T. Risk factors for the transmission of foot-and-mouth disease during the 2010 outbreak in Japan: a case-control study. BMC Vet Res. (2013) 9:150. 10.1186/1746-6148-9-150 23880398 PMC3724691

[23] Nurmoja I Mõtus K Kristian M Niine T Schulz K Depner K Viltrop A. Epidemiological analysis of the 2015–2017 African swine fever outbreaks in Estonia. Prev Vet Med. (2018) 181:104556. 10.1016/j.prevetmed.2018.10.001 30482617

[24] Benard HJ Stärk KDC Morris RS Pfeiffer DU Moser H. The 1997–1998 classical swine fever epidemic in The Netherlands—a survival analysis. Prev Vet Med. (1999) 42:235–48. 10.1016/S0167-5877(99)00078-1 10619158

[25] Elbers ARW Stegeman JA De Jong MCM. Factors associated with the introduction of classical swine fever virus into pig herds in the central area of the 1997/98 epidemic in the Netherlands. Vet Rec. (2001) 149:377–82. 10.1136/vr.149.13.377 11601514

[26] Elbers ARW Stegeman A Moser H Ekker HM Smak JA Pluimers FH. The classical swine fever epidemic 1997–1998 in the Netherlands: descriptive epidemiology. Prev Vet Med. (1999) 42:157–84. 10.1016/S0167-5877(99)00074-4 10619154

[27] Boender GJ Van Den Hengel R Van Roermund HJW Hagenaars TJ. The influence of between-farm distance and farm size on the spread of classical swine fever during the 1997–1998 epidemic in the Netherlands. PLoS One. (2014) 9:e95278. 10.1371/journal.pone.0095278 24748233 PMC3991596

[28] Postel A Becher P. Epidemiology, diagnosis and control of classical swine fever : recent developments and future challenges. Transbound Emerg Dis. (2018) 65:248–61. 10.1111/tbed.12676 28795533

[29] Donahue BC Petrowski HM Melkonian K Ward GB Mayr GA Metwally S Analysis of clinical samples for early detectionof classical swine fever during infection with low, moderate, and highly virulent strains in relation to the onset of clinical signs. J Virol Methods. (2012) 179:108–15. 10.1016/j.jviromet.2011.10.008 22036595

[30] Petrov A Blohm U Beer M Pietschmann J Blome S. Comparative analyses of host responses upon infection with moderately virulent classical swine fever virus in domestic pigs and wild boar. Virol J. (2014) 11:1–6. 10.1186/1743-422X-11-134 25073480 PMC4118204

[31] Stegeman A Elbers ARW Bouma A De Smit H De Jong MCM Transmission of classical swine fever virus within herds during the 1997–1998 epidemic in The Netherlands. Prev Vet Med. (1999) 42:201–18. 10.1016/S0167-5877(99)00076-8 10619156

[32] Kaden V Lange E Steyer H Bruer W Langner C. Role of birds in transmission of classical swine fever virus. J Vet Med Ser B Infect Dis Vet Public Heal. (2003) 50:357–59. 10.1046/j.1439-0450.2003.00670.x 14535936

[33] Truong QL Seo TW Yoon B Il Kim HC Han JH Hahn TW. Prevalence of swine viral and bacterial pathogens in rodents and stray cats captured around pig farms in Korea. J Vet Med Sci. (2013) 75:1647–50. 10.1292/jvms.12-0568 23892461 PMC3942947

[34] MAFF Interim Report on the Epidemiological Investigation for Classical Swine Fever Outbreaks in Japan. (2019). Available online at: https://www.maff.go.jp/j/syouan/douei/csf/pdf/index-281.pdf (accessed April 7, 2020).

[35] Stegeman JA Elbers ARW Bouma A De Jong MCM. Rate of inter-herd transmission of classical swine fever virus by different types of contact during the 1997-8 epidemic in The Netherlands. Epidemiol Infect. (2002) 128:285–91. 10.1017/S0950268801006483 12002547 PMC2869822

