# Supplementary material for: Epidemiology of Classical Swine Fever in Japan—A Descriptive Analysis of the Outbreaks in 2018–2019
Source: Front Vet Sci. 2020 Sep 22;7:573480. doi: 10.3389/fvets.2020.573480 (PMC7536261; doi:10.3389/fvets.2020.573480)
Supplement: Supplementary file 1 [file Table_1.DOCX]

**Supplementary Table 1.** Brief description of the questionnaire used in the epidemiological investigation

| Category | Items | Responses |
| --- | --- | --- |
| General Information | Type of farm | breeding / fattening / farrow-to-finish |
|  | Structure of pig houses | windowless / semi-windowless / with open windows |
|  | Number of pig houses | (number) |
|  | Number of animals | (number) |
| Pig flow | Introduction of pigs | name of original farm(s)  number of pigs introduced in the past 2 months  operation process at introduction |
|  | Shipment of pigs | name of destination farm(s)  number of pigs shipped in the past 2 months  operation process at shipment |
|  | Movement of pigs within the farm | From (id of a pig house) to (id of a pig house)  By transport cage / by foot / others (description) |
| Feedstuff | Type of feedstuff | commercial / others (description)  when recycled food is used:  details of the materials  details of heat-treatment |
| Farm management | Number of employees engaged | (number) |
|  | Daily operation process by each pig house | (description) |
|  | Visit(s) of veterinarian(s) | Visit(s) in the past 2 months (yes/no, details) |
|  | Vaccination/drug administration program(s) | (description) |
|  | Visit(s) of other people | (description on who/when/how) |
| Clinical course | First observation of abnormal status of pigs | (description) |
|  | Distribution of abnormal pigs in pig house(s) | (description on the status of pigs and the movement of abnormal pigs within the farm) |
| Biosecurity measures | At the entrance of the farm  Record of visitors  Disinfection of vehicles  Disinfection of ground  Disinfection of visitors/employees  Change of footwear  Change of clothes  At the entrance of pig houses  Disinfection of visitors/employees  Change of boots  Change of gloves and clothes | (yes/no and description) |
|  | Preventive measures against wild animals  Installation of fences  without electricity  with electricity  Use of bird-proof nets  Pest control at pig houses | (yes/no and description) |
